# Plasma Protein Biomarkers for Depression and Schizophrenia by Multi Analyte Profiling of Case-Control Collections

**DOI:** 10.1371/journal.pone.0009166

**Published:** 2010-02-11

**Authors:** Enrico Domenici, David R. Willé, Federica Tozzi, Inga Prokopenko, Sam Miller, Astrid McKeown, Claire Brittain, Dan Rujescu, Ina Giegling, Christoph W. Turck, Florian Holsboer, Edward T. Bullmore, Lefkos Middleton, Emilio Merlo-Pich, Robert C. Alexander, Pierandrea Muglia

**Affiliations:** 1 Neurosciences Centre of Excellence in Drug Discovery, GlaxoSmithKline R&D, Verona, Italy; 2 Discovery Statistics Europe, GlaxoSmithKline R&D, Harlow, United Kingdom; 3 Quantitative Sciences, GlaxoSmithKline R&D, Verona, Italy; 4 Genetics Division, Drug Discovery, GlaxoSmithKline R&D, Verona, Italy; 5 GSK Clinical Units, Addenbrooke's Hospital, Cambridge, United Kingdom; 6 Department of Psychiatry and Psychotherapy, Ludwig-Maximilians-University, Munich, Germany; 7 Max-Planck Institute of Psychiatry, Munich, Germany; 8 Behavioural and Clinical Neuroscience Institute, University of Cambridge, Cambridge, United Kingdom; 9 Genetics Division, Drug Discovery, GlaxoSmithKline R&D, Greenford, Middlesex, United Kingdom; University of Muenster, Germany

## Abstract

Despite significant research efforts aimed at understanding the neurobiological underpinnings of psychiatric disorders, the diagnosis and the evaluation of treatment of these disorders are still based solely on relatively subjective assessment of symptoms. Therefore, biological markers which could improve the current classification of psychiatry disorders, and in perspective stratify patients on a biological basis into more homogeneous clinically distinct subgroups, are highly needed. In order to identify novel candidate biological markers for major depression and schizophrenia, we have applied a focused proteomic approach using plasma samples from a large case-control collection. Patients were diagnosed according to DSM criteria using structured interviews and a number of additional clinical variables and demographic information were assessed. Plasma samples from 245 depressed patients, 229 schizophrenic patients and 254 controls were submitted to multi analyte profiling allowing the evaluation of up to 79 proteins, including a series of cytokines, chemokines and neurotrophins previously suggested to be involved in the pathophysiology of depression and schizophrenia. Univariate data analysis showed more significant p-values than would be expected by chance and highlighted several proteins belonging to pathways or mechanisms previously suspected to be involved in the pathophysiology of major depression or schizophrenia, such as insulin and MMP-9 for depression, and BDNF, EGF and a number of chemokines for schizophrenia. Multivariate analysis was carried out to improve the differentiation of cases from controls and identify the most informative panel of markers. The results illustrate the potential of plasma biomarker profiling for psychiatric disorders, when conducted in large collections. The study highlighted a set of analytes as candidate biomarker signatures for depression and schizophrenia, warranting further investigation in independent collections.

## Introduction

The search for peripheral markers for psychiatry disorders has been underway for many years, but, in spite of these efforts, a non-invasive blood-based test that could be used for diagnosis, or help to stratify patients based on disease subtype remains elusive [1]. Previous experimental attempts to generate reliable blood-derived markers have selected candidate biomarkers based on current models of disease pathogenesis. For example, studies in depression and schizophrenia have tested specific biomarkers based on the hypothesis of monoamine dysfunction, the immuno-inflammatory hypothesis, the neuroendocrine and the neuroplasticity hypothesis [2]. These investigations, usually relying on the selection of single readouts, have generated a number of putative biomarkers which still require replication in larger studies. Due to the presumed high level of etiologic heterogeneity and the overlap of dimensions across mood disorders and schizophrenia, standalone markers are unlikely to be specific or applicable on a wide scale and for a wide range of patients. For both depression and schizophrenia, monoamine-related markers have been studied with only partial success in terms of specificity of the marker, or replication of the findings. More recently, a number of studies have been carried out to evaluate the potential of neurotrophin markers such as BDNF in different psychiatric diseases, again resulting in evidence of association but also with many non-specific or conflicting findings (for some examples of discrepancies in schizophrenia and autism, see [2]). For depression, the most robust laboratory finding is probably the HPA dysfunction of depressed patients during acute phase, which has led to the development of neuroendocrine challenge tests as putative biomarkers with potential application in clinical context [3].

Another interesting line of research has focused on inflammatory-related markers, based on the evidence of reciprocal communication between immune and nervous systems and of altered immunological state in psychiatry diseases. For depression in particular a “cytokine hypothesis” has been developed that associates the dysregulation of the immuno-inflammatory system with the aetiology and the pathophysiology of major depressive disorder [4]. The theory is supported by the evidence of positive correlation between circulating levels of pro-inflammatory cytokines, acute phase proteins and chemokines and symptoms of depression and fatigue in humans and preclinical species. So far, the majority of studies in psychiatry have investigated small cytokine subsets, mainly monocytic pro-inflammatory cytokines such as IL-1, IL-6 and TNFα (see [5] for an analysis of studies of inflammatory markers in antidepressant treatment). Recently a larger panel of pro- and anti-inflammatory cytokines was measured in a case/control population of major depressive disorders (MDD) (49 cases and 49 controls) showing elevation of a number of additional cytokines not previously implicated in MDD, as well as of some previously untested chemokines [6]. These promising data are supportive of the application of wider profiling approaches to the identification of biomarker panels as diagnostic tools for the classification of psychiatric diseases.

Multi-analyte and array profiling techniques enable the simultaneous detection of hundreds of proteins with high sensitivity and accuracy and can be successfully applied to identify biomarkers (or clusters of biomarkers) that correlate with disease [7]. We describe here the application of a large protein profiling investigation for the identification of novel peripheral markers for depression and schizophrenia, based on a focused proteomic approach. A large number of plasma samples selected from well characterized psychiatric disease collections were submitted to protein profiling using a commercially available multi-analyte protein panel that contains a number of cytokines, chemokines, neurotrophins and hormones involved in pathways hypothesized to be involved in the pathophysiology of psychiatric diseases. The results obtained suggest that peripheral signatures for depression and schizophrenia may be identified by exploiting large clinical collections.

## Methods

### Patients

The present study was performed on plasma extracted from a subset of clinically well characterized cohorts of patients diagnosed with MDD and schizophrenia that were collected as part of larger genetic initiatives [8], [9].

The detailed clinical findings of the full MDD and schizophrenia cohorts have been reported elsewhere [8], [10]. All participants in these studies, which were approved by their respective local Ethics Boards, received a detailed description of the goals and funding of these studies and provided a written informed consent.

#### Major Depressive Disorders

A total of 1022 Caucasian patients with recurrent MDD were recruited at the Max-Planck Institute of Psychiatry in Munich, Germany and at two satellite recruiting hospitals (BKH Augsburg and Klinikum Ingolstadt) in the Munich area. Patients were evaluated using the semi-structured Schedule for Clinical Assessment in Neuropsychiatry (SCAN) instrument, administered by experienced research assistants who had received proper training at WHO Training and Research Centers. Patients were included in the study if they received a diagnosis of recurrent MDD (i.e. at least two separate episodes of depression) according to DSM-IV or ICD-10. Patients were excluded from the study if they had experienced mood incongruent psychotic symptoms, a lifetime history of intravenous drug use or diagnosis of drug dependency, depression secondary to alcohol or substance abuse or depression as clear consequence of medical illnesses or use of medications. Patients with co-morbid anxiety disorders, with the exception of obsessive compulsive and post traumatic stress disorders, were included. Patients with diagnosis of schizophrenia, schizoaffective disorders and other axis I disorders were excluded from the study.

#### Schizophrenia

Patients were assessed at the Ludwig-Maximilian University in Munich Germany. A total of 499 unrelated patients received a DSM-IV diagnosis of schizophrenia according to The Structured Clinical Interview for DSM-IV, SCID. Patients with a diagnosis of schizoaffective disorder or reported to be an intravenous drug user or with a lifetime diagnosis of dependency were excluded. All patients were at least 18 years of age and Caucasians. Detailed medical and psychiatric history interviews included the administration of Positive and Negative Symptom Scale (PANSS).

#### Controls

A total of 968 Caucasian non-affected individuals were recruited at the Max-Planck Institute of Psychiatry in Munich, Germany. All subjects were selected from a Munich-based community sample. They were screened for the presence of anxiety and mood disorders using the Composite International Diagnostic Screener [11]. Only individuals without mood and anxiety disorders and schizophrenia at screening were included.

### Sample Selection

To reduce the heterogeneity of patients and controls, plasma samples were selected from the available collection based on a number of available clinical and demographic criteria. Subjects with comorbidities for the following major medical conditions that could have an overt impact on the protein profile were excluded: cancer, multiple sclerosis, Parkinson's disease, rheumatoid arthritis, inflammatory bowel disorder (Chron's disease, ulcerative colitis), psoriasis, emphysema, chronic bronchitis, hayfever, diabetes type 1 (early onset), diabetes type 2 (late onset), heart attack, angina, stroke. In addition, patients older then 80 and with BMI <18.5 or >40 were excluded. In the depression and control data set, patients smoking more than twenty cigarettes/day were also excluded.

The demographics and main clinical characteristics of the samples submitted to protein profiling analysis are reported in Table 1.

**Table 1:** Demographic data of cases and controls submitted to biomarker analysis.

Parameter	Depression	Schizophrenia	Controls
Total number (N)	245	229	254
Gender (M/F)	78/167	115/114	81/173
Age (mean±SD)	53.2±14.3	37.8±10.7	48.9±14.2
BMI (mean±SD)	25.9±4.0	26.6±4.9	24.2±3.4
N of smokers (current/former/never)	38/32/177	130/34/65	42/88/124
N of treated*/untreated	225/20	205/24	1/253

*Treatments defined as monoamine re-uptake inhibitors, tryciclic antidepressants, monoamine oxidase inhibitors, mood stabilizers, typical and atypical antipsychotics.

### Plasma Samples

Blood (approximately 7.5 ml) was obtained by forearm vein and drawn in EDTA containing tubes for biomarker studies. The samples were centrifuged for 10 min at 4C and the resulting plasma aliquoted into Eppendorf tubes, which were frozen immediately at −80°C.

### Biomarker Profiling

A total of 741 samples were analysed by Rules Based Medicine, Inc with the Multi Analyte Profiling Human MAP, a quantitative, multiplexed immunoassay based on Luminex xMAP technology [12], [13] which measures a battery of analytes including chemokines, cytokines, hormones, growth factors, antigens and other protein markers. The following 79 analytes were assessed by using the Human MAP version 1.5: α-1 Antitrypsin; Adiponectin; α-2 Macroglobulin; AFP; Apolipoprotein A1; Apolipoprotein CIII; Apolipoprotein H; β-2 Microglobulin; BDNF; Complement 3; CA 125; CA 19–9; Calcitonin; CEA; CK-MB; CRP; EGF; ENA-78; Endothelin-1; Eotaxin; Erythropoietin; FABP; Factor VII; Ferritin; FGF basic; Fibrinogen; HGH; GM-CSF; GST; ICAM-1; IgA; IgE; IgM; IL-1 α; IL-1 β; IL-2; IL-3; IL-4; IL-5; IL-6; IL-7; IL-8; IL-10; IL-12p40; IL-12p70; IL-13; IL-15; IL-16; Insulin; Leptin; Lipoprotein (a); Lymphotactin; MCP-1; MDC; MIP-1 α; MIP-1 β; MMP-2; MMP-3; MMP-9; Myoglobin; PAI-1; PAP; PSA, Free; RANTES; Serum Amyloid P; Stem Cell Factor; SGOT; TBG; Tissue Factor; TIMP-1; TNF RII;TNF-α; TNF-β; Thrombopoietin; TSH; VCAM-1; VEGF; vWF (see Table S1, Supporting Information for additional information). Samples were processed and analyzed according to RBM standard operating procedures. All samples were stored at −80°C until tested. The samples were thawed at room temperature, vortexed, spun at 13,000×g for 5 minutes for clarification and volume was removed for MAP antigen analysis into a master microtiter plate. Using automated pipetting, an aliquot of each sample was introduced into one of the capture microsphere multiplexes of the Human Antigen MAP, thoroughly mixed and incubated at room temperature for 1 hour. Multiplexed cocktails of biotinylated, reporter antibodies for each multiplex were then added robotically and after thorough mixing, were incubated for an additional hour at room temperature. Multiplexes were developed using an excess of streptavidin-phycoerythrin solution which was thoroughly mixed into each multiplex and incubated for 1 hour at room temperature. The volume of each multiplexed reaction was reduced by vacuum filtration and the volume increased by dilution into matrix buffer for analysis. Analysis was performed in a Luminex 100 instrument and the resulting data stream was interpreted using proprietary data analysis software developed at Rules-Based Medicine (RBM Plate Viewer version 1.1.1). For each multiplex, both calibrators and controls were included on each microtiter plate. 8-point calibrators were run in the first and last column of each plate and 3-level controls were included in duplicate. Testing results were determined first for the high, medium and low controls for each multiplex to ensure proper assay performance. Unknown values for each of the analytes localized in a specific multiplex were determined using 4 and 5 parameter, weighted and non-weighted curve fitting algorithms included in the data analysis package. The plasma samples were run in duplicate and data reported back as concentrations (average of two independent measures), together with normative data such as least detectable dose (determined as the mean ±3 standard deviations of 20 blank readings) and lower assay limit (assay working sensitivity defined by the lowest concentration calibrator used for quantitation). Any value above the LDD will possess coefficients of variation (CV) less than 20%.

### Data Analysis

As each of the protein analytes is tested by a specific immunoassay, the resulting data have potentially different statistical properties and therefore different required data transformations prior to analysis. Automated procedures were considered unsuitable and appropriate transformations (none, square root or log) were selected by visual inspection of the group-wise distribution of the raw data for each single protein.

In addition, two different approaches were applied to recover part of the potential information carried out by the protein analytes that showed a significant percentage of samples with values below detection limit in at least one of the groups (depressed, schizophrenics and controls), with the percentage of “censored values” differing between the groups. In the first approach the value below the threshold of detection limit (“censored value”) was substituted by the most observed low value in the set. In a second approach, part of the censored signal was recovered by the mean-median imputation technique. This method exploits the property that the underlying data distribution is broadly symmetric after data transformation and hence imputation values are selected to force the mean and median values to coincide. Other additional methods including truncated normal maximum likelihood (Tobit's method) and Random Forests were also performed. Where minimum value imputation analysis was performed, our results were additionally validated by a stability analysis imputing changing fractions of the minimum values obtained for each response, which confirmed our main analysis.

### Statistical Tests

Statistical analysis was performed by a combination of univariate analysis, i.e. t-tests, analysis of variance (ANOVA) and multivariate analysis, including Principal Component Analysis (PCA), PCA followed by partial least squares discriminant analysis (PLS-DA), and a Random Forest algorithm. Statistical analyses were performed by using SIMCA-P+ version 11 (Umetrics AB, Umeå, Sweden) and SAS 9.1 (SAS Institute Inc., Cary, NC, USA) software. RF and LDA analysis were performed using R version 2.6.2 (R Foundation for Statistical Computing, Vienna, Austria).

For univariate methods, both ANOVA and non-parametric approaches were used in addition to standard transformation and rank transformations, minimum value and mean-median imputation. Similar approaches were followed for multivariate analysis leading to PCA and PLS-DA. Receiver Operating Characteristic (ROC) plots [14] were derived from linear discriminative analysis (LDA) based on the top findings from the PLS approach. Random forest (RF) algorithm [15] based on cross validation with a training set and a test set was applied to generate an independent multivariate discriminative model, for which ROC curves were also produced. For markers of interest, correlation with disease state was calculated by performing Spearman's correlation tests.

## Results

### Univariate Analysis

Univariate analysis was carried out to identify single protein signals that are associated with disease status and to establish information and methodology applicable to multivariate approach in subsequent analysis.

Three different approaches were used: i) t-tests and standard ANOVA on log or square root ad-hoc transformed protein values, and replacement of values below detection limit by the lowest valid observed value; ii) ANOVA after first-ranking data transformation; iii) a non-parametric version of the t-test (Wilcoxon). All three approaches gave similar results (not shown) which led us to use the first two data transformation approaches for subsequent multivariate analysis.

Results from t-tests showed strong differences between the control and both disease groups, with larger differences observed for schizophrenia cases. Analysis of parametric, non-parametric and rank transformation results shows that there are many more significant p-values than what would be expected by chance (see Supporting Information, Figure S1).

**Figure 1:**
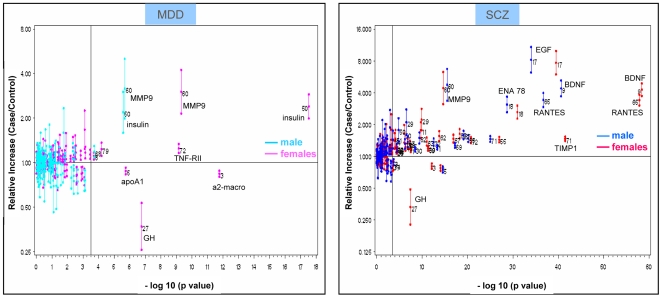
Relative change of protein markers in MDD or schizophrenia against their p-values. Plot of relative changes for measured analytes in depression (a) and schizophrenia (b) against their p-values. The Y axis reports the relative increase (or decrease) as the ratio (and the confidence interval) based on analysis of log-transformed data from cases/controls. Reference vertical line corresponds to p-value threshold at a 5% significance level, after correction for multiple testing. Male and females are computed and reported separately.

Figure 1 shows the relative difference (fold changes with confidence intervals) for each single protein when comparing samples from MDD versus controls and samples from schizophrenia versus controls, by gender. To obtain a common scale, all results presented are based upon log transformed data. As it can be seen, many protein differences have statistical significance well above the highly conservative Bonferroni correction threshold (represented by the vertical line, i.e. a p-value threshold of 5% significance level corrected for multiple testing), in particular for schizophrenia. For depression, the analyte that showed the highest difference between cases and control was insulin (in particular for female subjects). A significant and consistent increase was also observed for MMP-9, with p-values ranging between 10^−10^ and 10^−20^. For schizophrenia, BDNF, Rantes and EGF gave the strongest signals, with p-values ranging approximately between 10^−30^ and 10^−60^.

**Figure 2:**
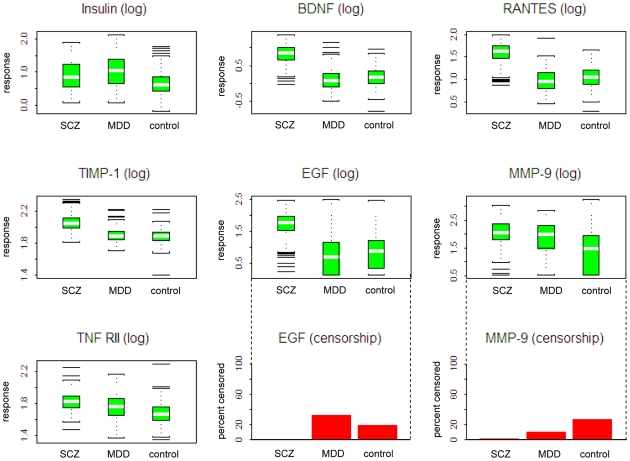
Plasma protein markers with highest significance in MDD and schizophrenia. Box plots of individual analytes with high univariate significance. Where data below detection limit were present (see bar charts on bottom row for EGF and MMP-9) they are replaced by the lowest observed value to generate the box plots. The bracketed values in the titles refer to the data transformation, if applied, and the sequence number in the original dataset. The white line corresponds to the median, whilst the full box represents the central 50%.

Full data (p-values by analyte and by group, from LSD test on transformed data) are included in Table S2 in Supporting Information. Figure 2 shows the combined plot (for both MDD and schizophrenia) corresponding to some of the analytes with highly statistically significant findings in both comparisons, according to univariate analysis. For some protein analytes, the observed change is clearly more marked in one of the disease groups, such as in the case of BDNF and EGF for schizophrenia and insulin for depression.

A formal analysis was also performed on additional demographic covariates to investigate the potential occurrence of stratification effects impacting the above results, due to the differences in gender ratio or mean BMI and age between groups. Results of this investigation are shown in Supporting Information, Table S3, which reports all p-values obtained for disease effect, for demographic effects (age, gender and BMI), for disease effect after inclusion of demographic covariates, and disease/covariate interaction. The above analysis indicates that some of the putative MDD- or schizophrenia-associated markers display also significant association with demographic covariates, such as insulin with BMI or BDNF with age. However, the comparison of simple p values for disease effects with p values obtained after fitting covariate effects indicates that there are no major deviations from the original results, at least for the top findings (see also Supporting Information, Figure S2). For instance, age effects do not appear to modify substantially the highly significant associations found for BDNF, RANTES, EGF, TIMP-1, ENA-78 and MDC levels with schizophrenia. For depression, the insulin elevation in patients compared with controls remains highly significant after fitting BMI, as well as across different BMI ranges (not shown). Further analyses were carried out for insulin, to assess potential dietary and site confounders that might have affected the finding of its elevation in depressed patients. By adjusting for time since last meal (minutes) and blood glucose levels available from the clinical chemistry panel, the observed elevation remains highly significant (see Supporting Information, Figure S3).

### Multivariate Analysis

Multivariate analysis was also carried out to explore correlations within the dataset, and to identify whether multiple analytes could increase the discrimination between cases and controls. The analysis was performed in two stages: i) principal components analysis (PCA) for unsupervised analysis of the full dataset, aimed at determining whether a multivariate signal was present; ii) partial least squares discriminant analysis (PLS-DA) to help identifying the identity of the proteins responsible for the separation.

**Figure 3:**
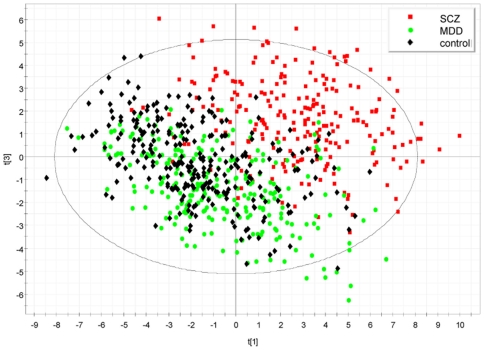
PCA plot showing the separation of schizophrenia samples from controls and MDD. PCA plot obtained by using SIMCA, where the 1st and 3rd components of the model (t[1] and t[3], respectively) are shown. The graph is obtained by replacing values below detection limit with the lowest value measured for each protein (conservative approach).

Figure 3 shows a PCA plot obtained by using SIMCA. The graph is obtained by the pragmatic approach of replacing values below detection limit with the lowest robust value measured for each protein, and results are in close agreement with those obtained using other approaches (e.g. with rank observations, data not shown). Similar to the results of the univariate analysis, a strong separation can be observed, in particular for schizophrenia samples.

**Figure 4:**
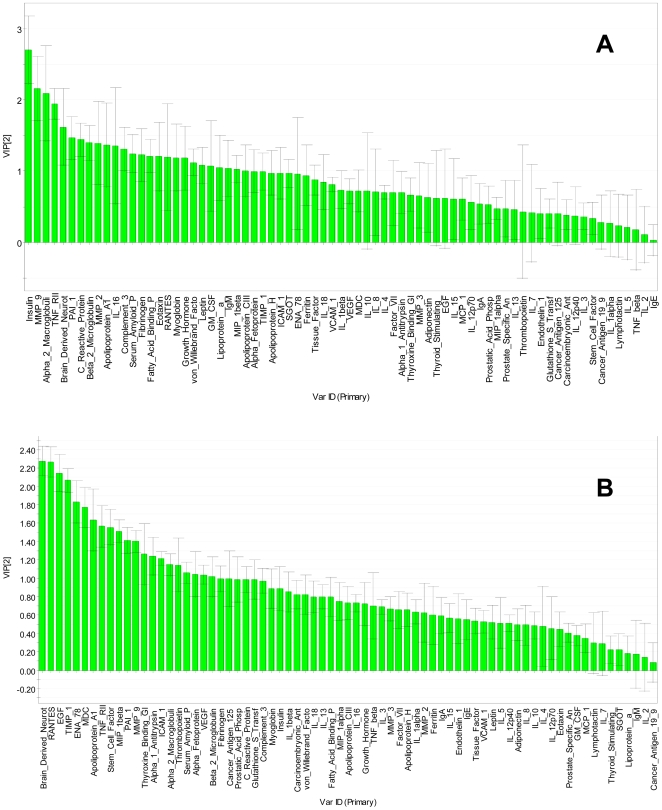
Contribution from each individual marker to case-control separation from PLS discrimination analysis. Variable Importance of Information (VIP) plot ranking markers for their contribution to case-control separation from PLS discrimination analysis. A: MDD; B: schizophrenia. Larger values on the left indicate more important contributions.

It should be noted that the above graph were produced by PCA without previous disease classification information, in contrast to partial least squares (PLS) approach, which is known to split classified groups even from random data sets. Having established a separation by PCA, the step of PLS-discriminant analysis was used solely to compute a series of scores (variable importance in the projections, or VIPs) to assess the contribution of individual proteins to these dimensions. The two graphs in Figure 4 (a,b) show the contribution of information from each individual variable to the overall control-depression and control-schizophrenia separation by PLS discrimination analysis.

**Figure 5:**
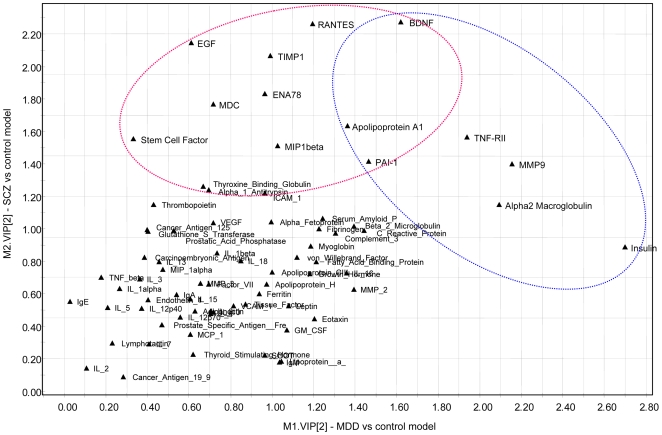
Comparing marker contributions to case-control separation for MDD versus schizophrenia. The Variable Importance of Contribution (VIP) from PLS-DA for each analyte is plotted on X axis for MDD and on the Y axis for schizophrenia. The overlap between the two groups of markers highlights findings that are in common between the two disease groups.

In Figure 5, the contribution of each single analyte to the separation of disease from control samples (VIP) are plotted for schizophrenia and depression on the y- and x- axis, respectively, to provide a visual representation of the relative specificity of the findings. The analytes highlighted in the box could be considered as the best informative or diagnostic set to discriminate disease from controls in the two categories. Proteins falling in the overlapping region may contribute to the separation but can be expected to be less specific markers.

**Figure 6:**
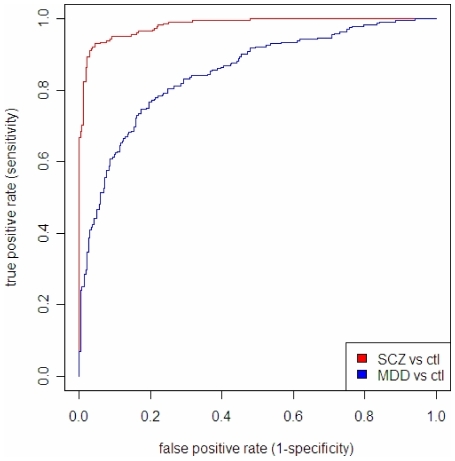
Receiver-operating characteristic (ROC) plot derived from linear discriminative analysis (LDA) based on the top findings from the PLS approach. ROC plot of sensitivity (True Positive Rate, Y-axis) versus 1 – specificity (False Positive Rate, X-axis) based on a Linear discrimination model (LDA) built upon the 10 markers with the highest contribution as determined by PLS-DA.

To assess the capability of our diagnostic set of marker to discriminate correctly between cases and controls, we have derived ROC plots [14] based on a linear discrimination analysis model (LDA) built upon the 10 markers with the highest contribution as determined by PLS-DA (see VIP plots). As it can be seen in Figure 6, the selected analytes are showing a good degree of selectivity/specificity pair for MDD/control and a superior discriminative power for SCZ from controls, with a true positive rate greater than 90% when setting the criterion for false positive at 5% (specificity >95%). To corroborate the above findings by an independent method, we have applied to the data a random forest (RF) algorithm, which included variable selection and cross validation with a training set and a test set. The discriminative model generated by RF and the corresponding ROC curves (Figure S4, Supporting Information) basically confirm the findings obtained by more traditional multivariate PCA and PLS analysis.

**Figure 7:**
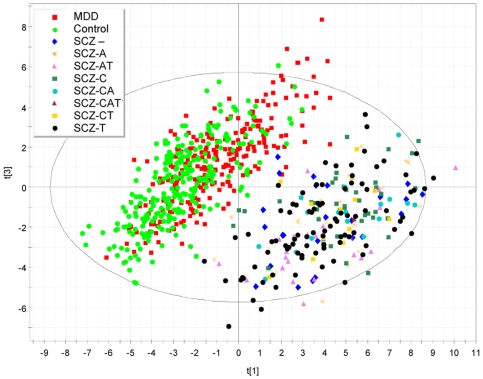
PCA plot showing the lack of separation of untreated from treated schizophrenics by plasma profiling. PCA plot obtained by SIMCA coded according with the different medications for schizophrenia cases (“C” indicates treatment with clozapine, “A” treatment with other atypical antipsychotics, “T” indicates treatment with typical antipsychotics, “-“ indicates untreated subjects). The dark blue (untreated, diamond) schizophrenics samples do not separate out from the whole schizophrenic group. t[1] and t[3] represent the 1st and 3rd component of the PCA model.

A number of ad hoc tests were then performed in order to verify the impact of some covariates on the separations observed in PCA. To assess the possibility that potential confounders could be responsible for the strong separation observed between schizophrenics and controls we have analysed PCA plots generated by the multivariate analysis in the context of additional parameters, including psychotropic drug treatment. The data in Figure 7, for instance, suggest that the modulation of the protein profile in samples from schizophrenic patients appears to be independent from treatment, as non-medicated patients could not be separated from patients medicated with different antipsychotics, neither a specific antipsychotic treatment group was observed.

### Correlation Analysis

The potential correlation between the levels of the identified markers and disease severity was assessed for the schizophrenia subset, for which PANSS data obtained at the sampling visit were available for all patients. Results obtained by performing a Spearman's correlation test on analytes identified by the previous approach (see Figure S5, Supporting Information), particularly in view of the large sample sizes used, do not provide strong evidence of a significant correlation of the protein values with the severity scale, further suggesting that the signal identified are likely to be trait markers.

## Discussion

The study reported here is one of the first focused clinical proteomic investigations carried out in a large clinical population of psychiatric patients, based on the profiling of a number of proteins belonging to pathways previously shown to be involved in the pathophysiology of either depression or schizophrenia (such as growth factors, cytokines) or previously untested. The results obtained have highlighted a number of differences between cases and controls which is well above what could be expected under the null hypothesis, with several protein analytes that appeared to be specifically modulated in one of the two disease groups. As shown by the univariate analysis, the significance of the observed differences is higher for schizophrenia than depression, resulting with a very high discriminative power of the panel for SCZ from controls as shown by the ROC plots.

In depression, univariate analysis highlighted a significant difference in particular for insulin and matrix metallo-proteinase 9 (MMP-9), which was also highlighted by the multivariate approach. Insulin was the marker with the highest statistical significant finding, shown to be increased in MDD cases compared to controls. When insulin data were stratified by glucose and time from last meal to compensate for potential post-prandial confounder effects and the effects of glucose-insulin homeostasis, the results remained statistically significant. The observed data are consistent with the observation that depression is frequently linked with insulin resistance: reduced glucose utilization and elevated insulin secretion following glucose administration have been shown in depressed patients [16], [17]. The high comorbidity between type 2 diabetes and depression [18] and the strong association between depression and metabolic syndrome [19] are further justifications to support the above hypothesis. More recently, evidence of a bidirectional relation between hypercortisolemia in depressed patients and poor glycemic control was provided [20]. The studies reported so far highlight an impairment of insulin sensitivity in depressed patients which is state-dependent [21], and no detectable difference in 24 h mean glucose and insulin levels from healthy controls [20]. However, due to the increased statistical power of the current study, one cannot exclude that the difference in physiological insulin secretion observed is reflecting an underlying trait of depression associated with impaired glycemic control. The high significance of the finding remained after the inclusion of BMI as a covariate (see Supporting Information) and after inclusion of potential dietary confounders suggesting that the increase in physiological insulin levels were not driven primarily by dietary or BMI differences across sites or between cases and controls.

Our analysis also revealed that members of the extracellular proteolytic system, composed of matrix metalloproteinases (MMPs) and their endogenous tissue inhibitors (TIMPs), were modulated in the two disease groups (increased MMP-9 levels, and, to a lesser extent, decreased MMP-2 levels in MDD; increased TIMP-1 levels and MMP-9 levels in schizophrenia). MMPs display a key role in central nervous system as they are able to process several proteins crucial for synaptogenesis, synaptic plasticity, and long-term potentiation (LTP) [22]. MMP-9 was specifically shown to regulate synaptic plasticity in the hippocampus by gain- and loss-of-function studies on LTP in vitro [23], [24]. Experimental evidence supports the hypothesis that the MMP/TIMP ratio modulates neuronal plasticity in learning and memory processes [25]. TIMP-1, which binds to MMP-9 and regulate its activity, was indeed shown to be able to prevent MMP-9-dependent late LTP in the rat medial PFC [26]. MMPs and TIMPs have been also investigated as potential markers for dementia, resulting with the identification of altered plasma levels of MMP-9 and TIMP-1 in Alzheimer's Disease and vascular dementia, respectively [27].

MMPs have many other properties, including the ability to modulate cytokines and growth factors (such as TNF-alpha and BDNF among others) by processing their proforms into active forms [22]. Interestingly, similar to what we have found in our case, patients with metabolic syndrome also display increased circulating concentrations of pro-MMP-9, MMP-8, and TIMP-1, which were associated with increased concentrations of pro-inflammatory mediators and adhesion molecules [28]. Of note, our supplemental data show a positive association of TIMP-1 levels with age in MDD patients and controls, which is not detected in the schizophrenia group which has elevated levels in spite of a lower mean age with respect to controls.

For MDD, several studies suggest a link between circulating cytokines and depressive episodes. Increased circulating levels of pro-inflammatory cytokine, acute phase proteins and chemokines are known to be associated to symptoms of depression and fatigue in humans and preclinical animal species [4], [29], [30]. Increased circulating levels of IL-6 and TNF-α were described in a consistent susceptible population of patients suffering from Major Depressive Disorders during the symptomatic episode [31]–[35] and correlation between the high levels of IL-6 in the morning and depressive symptoms were found in MDD patients by Alesci et al. [36]. Successful antidepressant treatments of MDD episodes with SSRIs or TCAs are associated to the reduction of circulating cytokine levels, in particular TNF-α [37], [38] and IL-6 [39]. A recent meta-analysis on inflammatory markers in depressed patients has confirmed a consistent positive association between depression and IL-6, and IL-1 and the acute phase protein CRP levels in peripheral blood [40]. In addition, it was shown that major depression is characterized by an acute phase response, with elevated levels of positive acute phase proteins [41], [42]. Accordingly, we have found a number of acute-phase proteins (α2-macroglobulin, C-reactive protein and β2-microglobulin) that contributed to the separation between cases and controls. However, beside a significant signal obtained by increased TNF-RII, consistent with recent findings [43]–[45], we did not observe pro- and anti-inflammatory proteins amongst the analytes with the highest contribution to the separation. This may be due to the fact that IL-6 and TNF-α, the most significant findings according to the literature, had too many missing values in our dataset to become significant. A recent analysis of inflammatory markers in MDD patients using a cytokine panel has confirmed the elevation of pro-inflammatory interleukins and highlighted abnormalities in additional factors, such as MIP-1 and eotaxin, not previously implicated in MDD [6]. When comparing our data with the above results, we found little degree of overlap. The above observations may be reconciled by considering that the elevation in these pro-inflammatory cytokines is thought to be symptom- or state-related, whilst most MDD cases in the present investigation were sampled outside the acute episode, resulting with likely markers for trait.

From the analysis of the data from schizophrenia sample, it can be observed that the strong separation from controls is due to protein analytes belonging to the growth factors and neurotrophin family, such as BDNF, EGF or stem cell factor, and to a lesser extent from member of the chemokine/cytokine family. Neurotrophin/growth factor levels were previously reported to be altered in samples from schizophrenics with respect to control samples in both peripheral and central tissues. For EGF, decreased levels were found in serum from schizophrenic patients [46], [47], even though a previous study has failed to show significant differences between 40 cases and 40 controls [48]. For BDNF, previous studies in schizophrenia have produced mixed results, with evidence of increased, decreased, or no change in serum or plasma BDNF level [47], [49]–[52]. It should be noted that the sample size of the current study is one order of magnitude larger than the ones used in previous studies. However we cannot rule out potential stratification (for example differences in mean age) or site effects as potential confounders in our study, and further investigation in separate collections are warranted. In contrast, for MDD a small but significant overall decrease of BDNF was found, in line with most clinical observations reported so far [53]. Among proteins belonging to other pathways, a number of chemoattractants were found to be modulated in schizophrenia samples, i.e. ENA-78 (Epithelial Neutrophil Activating Peptide-78, a recently discovered chemoattractant and activator for neutrophils, belonging to the IL-8 subgroup of the C-X-C family of chemokines); CCL5 or Rantes (which functions as a chemo-attractant for blood monocytes, memory T helper cells and eosinophils; it is one of the natural ligands for the chemokine receptor CCR5); MDC (Macrophage-derived chemokine or CCL22, a functional ligand for the CC chemokine receptor and a novel chemoattractant for monocytes, monocyte-derived dendritic cells, and natural killer cells). Interestingly, alterations in peripheral chemokine levels have been recently observed in bipolar and schizophrenia patients [54], [55].

All the above analytes contributed to the strong separation observed between schizophrenic cases and controls, which appears to be unrelated to treatment regimen. In addition, we have found some degree of correlation between analytes that significantly contributed to the separation between schizophrenic and control samples (not shown), suggesting that the difference in levels between disease and control samples maybe arise from a modulation of a common biological mechanism. While one cannot completely rule out that the observed changes are arising from technical differences between sites in sampling procedures for instance, it is difficult to understand how these site effects could specifically impact only on a subset of correlated analytes. Another variable to consider in particular for the schizophrenia group is the significantly higher percentage of active smokers with respect to controls. However, the plasma levels of the top findings (such as BDNF, RANTES, TIMP-1, EGF) was still altered in non-smokers, and an obvious correlation between the number of cigarettes per day with the marker values in the smoker subset could not be found (data not shown). After ruling out medication effects, and considering that we have found no significant correlations with PANSS values, the observed changes are more likely to reflect an impairment in pathways that underlie a characteristic trait for schizophrenic patients (such as neurodevelopmental abnormalities or aberrant plasticity pathways), or could reflect a chronic systemic (inflammatory) response.

In conclusion, by applying multi analyte profiling on a large collection of MDD and schizophrenia, we have identified a pattern of analytes that appear to discriminate cases from controls. The analytes that have contributed to the separation belong to pathways or mechanisms previously known to be involved in the pathophysiology of the disease (e.g. neurotrophins in schizophrenia) or associated to comorbid states (e.g. insulin resistance in depression). The validity of the findings is supported by the use of alternative and complementary multivariate statistical approaches which resulted with similar output. However, there are a number of limitations of the current study that need to be taken into account. The primary objective of the collection was the conduction of genetic association studies. The depression diagnosis is lifetime and the subjects were in different active disease states when the samples were taken or the interviews conducted, and data on the patients' mood state were not available for analysis. Accordingly, the study has a limited capacity to identify state or severity markers but rather biological markers underlying different disease traits. Cases were recruited from three different study sites in the same region whereas controls were recruited from one of the sites, and we have examined differences in the average time of sampling from the last meal at the different study centres which in principle could affect some analytes influenced by diet/metabolism e.g. insulin, blood glucose, leptin. An additional limitation is the difference in some of the demographic parameters between the schizophrenic group and the MDD and control groups (in particular mean age and gender ratio), though the results remained significant when fitting the specific effect of single covariates. Finally, all proteins were measured in plasma and, although changes in peripheral levels may partly reflect changes occurred in the brain, we can only speculate about the physiological role of the identified markers in the central nervous system.

Notwithstanding the above potential limitations, this study suggests that disease signatures derived from large scale analysis of blood samples from psychiatric patients may exist and could be detected by using large and well characterized sample sets. For schizophrenia, more significant differences have been detected, which would need to be replicated in a separate cohort to assess the impact of potential stratification or site effects. Putative marker sets for disease trait have been identified, that could help to delineate homogeneous depression or schizophrenia subgroups based on biological evidence and, in the long term, generate objective criteria for patient selection. The insight generated from the current analysis should drive the design of a biomarker panel to be applied to longitudinal clinic studies for antidepressant and antipsychotics. In this way, it would be possible to establish if any of the identified biomarkers also correlate with clinical improvement, setting the basis for the development of a biomarker panel to assess disease severity. Our results call for further investigation in other accessible tissues (such as the cerebrospinal fluid) or in other psychiatric disorders, as well as for replication from independent groups.

## Supporting Information

Table S1 List of markers assessed by the multi-analyte panel MAP 1.5 and their Least Detectable Dose.
(0.11 MB PDF)

Table S2 Univariate analysis full data set. The table reports p-values by analyte and by disease group, obtained from Least Significant Difference test on transformed data analysis from MDD plus controls or SCZ plus controls; minimum value imputation approach (see Materials and Methods). Standard errors and difference on transformed scale are reported. For log transformed data, the difference corresponds to the log of the ratio between cases and controls.
(0.04 MB XLS)

Table S3 Effect of age, BMI and gender as covariates. The table reports the results from a more formal analysis performed on possible demographic covariates to verify if differences in BMI, age or gender ratio could impact the results obtained by the disease group analysis. The analysis aimed at verifying if (i) the clinical parameters chosen where statistically significant and (ii) if their inclusion significantly changed the results for the disease groups. Statistical interactions were also tested in these models. Worksheet 1: p values for demographics (gender, age and BMI); from separate ANOVA analysis based on full dataset (MDD, SCZ and controls). Worksheets 2–11: simple p values for disease group (t-test), p values for demographics, p values for disease group after inclusion of demographics, demographic by group interaction; from ANOVA on MDD plus controls or SCZ plus controls. More specifically: effect of gender (Worksheet 2,3); effect of age, linear model (Worksheet 4,5); effect of age, quadratic model to account for potential non-linearity (Worksheet 6,7); effect of BMI, linear model (Worksheet 8,9); effect of BMI, quadratic model to account for potential non-linearity (Worksheet 10,11)
(0.17 MB XLS)

Figure S1 Non-parametric (a) and rank transformation (b) results referenced against analysis of variance with minimum value imputation. The observed p-values strongly deviate from the expected p-values that would be expected by chance, both for depression (MDD) and schizophrenia (SCZ) samples.
(0.27 MB PDF)

Figure S2 Simple p values vs p values after covariate analysis. Correlations between simple p values and p values after inclusion of covariates in the analysis.
(0.21 MB PDF)

Figure S3 Effect of dietary confounders on insulin levels (depression samples).
(0.10 MB PDF)

Figure S4 Results from Random Forest (RF) algorithm. Discriminative models based on cross validation with a training set and a test set generated by RF and relative ROC curves.
(0.28 MB PDF)

Figure S5 Correlation with clinical severity (schizophrenia samples). Results from Spearman's correlation test run between protein levels in the plasma and PANSS value for schizophrenic patients. The central dots are the correlations, and the horizontal lines their 95% confidence intervals. The twenty analytes with the highest correlation are shown.
(0.07 MB PDF)

## References

[1] Connor TJ, Leonard BE, Preskorn SH, Feighner JP, Stanga C, Ross R (2004). Biological markers for Depression..

[2] Domenici E, Muglia P (2007). The search for peripheral markers in psychiatry by genomic and proteomic approaches.. Exp Opin Med Diagn.

[3] Ising M, Horstmann S, Kloiber S, Lucae S, Binder EB (2007). Combined Dexamethasone/Corticotropin Releasing Hormone Test Predicts Treatment Response in Major Depression-A potential Biomarker?. Biol Psychiatry.

[4] Raison CL, Capuron L, Miller AH (2006). Cytokines sing the blues: inflammation and the pathogenesis of depression.. Trends Immunol.

[5] Miller AH, Maletic V, Raison CL (2009). Inflammation and its discontents: the role of cytokines in the pathophysiology of major depression.. Biol Psychiatry.

[6] Simon NM, McNamara K, Chow CW, Maser RS, Papakostas GI (2008). A detailed examination of cytokine abnormalities in Major Depressive Disorder.. Eur Neuropsychopharmacol.

[7] Ray S, Britschgi M, Herbert C, Takeda-Uchimura Y, Boxer A (2007). Classification and prediction of clinical Alzheimer's diagnosis based on plasma signaling proteins.. Nat Med.

[8] Muglia P, Tozzi F, Galwey NW, Francks C, Upmanyu R (2008). Genome-wide association study of recurrent major depressive disorder in two European case-control cohorts.. Mol Psychiatry.

[9] Need AC, Ge D, Weale ME, Maia J, Feng S (2009). A genome-wide investigation of SNPs and CNVs in schizophrenia.. PLoS Genet.

[10] Van den Oord EJ, Rujescu D, Robles JR, Giegling I, Birrell C (2006). Factor structure and external validity of the PANSS revisited.. Schizophr Res.

[11] Wittchen HU, Lachner G, Wunderlich U, Pfister H (1998). Test-retest reliability of the computerized DSM-IV version of the Munich-Composite International Diagnostic Interview (M-CIDI).. Soc Psychiatry Psychiatr Epidemiol.

[12] Krishhan VV, Khan IH, Luciw PA (2009). Multiplexed microbead immunoassays by flow cytometry for molecular profiling: Basic concepts and proteomics applications.. Crit Rev Biotechnol.

[13] Chowdhury F, Williams A, Johnson P (2009). Validation and comparison of two multiplex technologies, Luminex® and Mesoscale Discovery, for human cytokine profiling.. J Immunol Methods.

[14] Fawcett T (2006). An introduction to ROC analysis.. Pattern Recogn Lett.

[15] Breiman L (2001). Random Forests.. Machine Learning.

[16] Tashiro A, Hongo M, Ota R, Utsumi A, Imai T (1997). Hyper-insulin response in a patient with depression. Changes in insulin resistance during recovery from depression.. Diabetes Care.

[17] Winokur A, Maislin G, Phillips JL, Amsterdam JD (1988). Insulin resistance after oral glucose tolerance testing in patients with major depression.. Am J Psychiatry.

[18] Katon WJ (2008). The comorbidity of diabetes mellitus and depression.. Am J Med.

[19] Dunbar JA, Reddy P, vis-Lameloise N, Philpot B, Laatikainen T (2008). Depression: an important comorbidity with metabolic syndrome in a general population.. Diabetes Care.

[20] Weber B, Schweiger U, Deuschle M, Heuser I (2000). Major depression and impaired glucose tolerance.. Exp Clin Endocrinol Diabetes.

[21] Okamura F, Tashiro A, Utumi A, Imai T, Suchi T (2000). Insulin resistance in patients with depression and its changes during the clinical course of depression: minimal model analysis.. Metabolism.

[22] Ethell IM, Ethell DW (2007). Matrix metalloproteinases in brain development and remodeling: synaptic functions and targets.. J Neurosci Res.

[23] Bozdagi O, Nagy V, Kwei KT, Huntley GW (2007). In vivo roles for matrix metalloproteinase-9 in mature hippocampal synaptic physiology and plasticity.. J Neurophysiol.

[24] Nagy V, Bozdagi O, Matynia A, Balcerzyk M, Okulski P (2006). Matrix metalloproteinase-9 is required for hippocampal late-phase long-term potentiation and memory.. J Neurosci.

[25] Chaillan FA, Rivera S, Marchetti E, Jourquin J, Werb Z (2006). Involvement of tissue inhibition of metalloproteinases-1 in learning and memory in mice.. Behav Brain Res.

[26] Okulski P, Jay TM, Jaworski J, Duniec K, Dzwonek J (2007). TIMP-1 abolishes MMP-9-dependent long-lasting long-term potentiation in the prefrontal cortex.. Biol Psychiatry.

[27] Lorenzl S, Buerger K, Hampel H, Beal MF (2008). Profiles of matrix metalloproteinases and their inhibitors in plasma of patients with dementia.. Int Psychogeriatr.

[28] Goncalves FM, Jacob-Ferreira AL, Gomes VA, Casella-Filho A, Chagas AC (2009). Increased circulating levels of matrix metalloproteinase (MMP)-8, MMP-9, and pro-inflammatory markers in patients with metabolic syndrome.. Clin Chim Acta.

[29] Dunn AJ, Swiergiel AH, de BR (2005). Cytokines as mediators of depression: what can we learn from animal studies?. Neurosci Biobehav Rev.

[30] Elenkov IJ, Iezzoni DG, Daly A, Harris AG, Chrousos GP (2005). Cytokine dysregulation, inflammation and well-being.. Neuroimmunomodulation.

[31] Mikova O, Yakimova R, Bosmans E, Kenis G, Maes M (2001). Increased serum tumor necrosis factor alpha concentrations in major depression and multiple sclerosis.. Eur Neuropsychopharmacol.

[32] Kubera M, Kenis G, Bosmans E, Zieba A, Dudek D (2000). Plasma levels of interleukin-6, interleukin-10, and interleukin-1 receptor antagonist in depression: comparison between the acute state and after remission.. Pol J Pharmacol.

[33] Maes M, Bosmans E, de JR, Kenis G, Vandoolaeghe E (1997). Increased serum IL-6 and IL-1 receptor antagonist concentrations in major depression and treatment resistant depression.. Cytokine.

[34] Maes M, Meltzer HY, Bosmans E, Bergmans R, Vandoolaeghe E (1995). Increased plasma concentrations of interleukin-6, soluble interleukin-6, soluble interleukin-2 and transferrin receptor in major depression.. J Affect Disord.

[35] Hestad KA, Tonseth S, Stoen CD, Ueland T, Aukrust P (2003). Raised plasma levels of tumor necrosis factor alpha in patients with depression: normalization during electroconvulsive therapy.. J ECT.

[36] Alesci S, Martinez PE, Kelkar S, Ilias I, Ronsaville DS (2005). Major depression is associated with significant diurnal elevations in plasma interleukin-6 levels, a shift of its circadian rhythm, and loss of physiological complexity in its secretion: clinical implications.. J Clin Endocrinol Metab.

[37] Tuglu C, Kara SH, Caliyurt O, Vardar E, Abay E (2003). Increased serum tumor necrosis factor-alpha levels and treatment response in major depressive disorder.. Psychopharmacology (Berl).

[38] Narita K, Murata T, Takahashi T, Kosaka H, Omata N (2006). Plasma levels of adiponectin and tumor necrosis factor-alpha in patients with remitted major depression receiving long-term maintenance antidepressant therapy.. Prog Neuropsychopharmacol Biol Psychiatry.

[39] Lanquillon S, Krieg JC, Bening-Abu-Shach U, Vedder H (2000). Cytokine production and treatment response in major depressive disorder.. Neuropsychopharmacology.

[40] Howren MB, Lamkin DM, Suls J (2009). Associations of depression with C-reactive protein, IL-1, and IL-6: a meta-analysis.. Psychosom Med.

[41] Van HF, Wauters A, Vandoolaeghe E, Neels H, Demedts P (1996). Lower total serum protein, albumin, and beta- and gamma-globulin in major and treatment-resistant depression: effects of antidepressant treatments.. Psychiatry Res.

[42] Maes M, Wauters A, Neels H, Scharpe S, Van GA (1995). Total serum protein and serum protein fractions in depression: relationships to depressive symptoms and glucocorticoid activity.. J Affect Disord.

[43] Himmerich H, Fulda S, Linseisen J, Seiler H, Wolfram G (2008). Depression, comorbidities and the TNF-alpha system.. Eur Psychiatry.

[44] Himmerich H, Fulda S, Linseisen J, Seiler H, Wolfram G (2006). TNF-alpha, soluble TNF receptor and interleukin-6 plasma levels in the general population.. Eur Cytokine Netw.

[45] Grassi-Oliveira R, Brietzke E, Pezzi JC, Lopes RP, Teixeira AL (2009). Increased soluble tumor necrosis factor-alpha receptors in patients with major depressive disorder.. Psychiatry Clin Neurosci.

[46] Futamura T, Toyooka K, Iritani S, Niizato K, Nakamura R (2002). Abnormal expression of epidermal growth factor and its receptor in the forebrain and serum of schizophrenic patients.. Mol Psychiatry.

[47] Ikeda Y, Yahata N, Ito I, Nagano M, Toyota T (2008). Low serum levels of brain-derived neurotrophic factor and epidermal growth factor in patients with chronic schizophrenia.. Schizophr Res.

[48] Hashimoto K, Shimizu E, Komatsu N, Watanabe H, Shinoda N (2005). No changes in serum epidermal growth factor levels in patients with schizophrenia.. Psychiatry Res.

[49] Gama CS, Andreazza AC, Kunz M, Berk M, Belmonte-de-Abreu PS (2007). Serum levels of brain-derived neurotrophic factor in patients with schizophrenia and bipolar disorder.. Neurosci Lett.

[50] Grillo RW, Ottoni GL, Leke R, Souza DO, Portela LV (2007). Reduced serum BDNF levels in schizophrenic patients on clozapine or typical antipsychotics.. J Psychiatr Res.

[51] Shimizu E, Hashimoto K, Watanabe H, Komatsu N, Okamura N (2003). Serum brain-derived neurotrophic factor (BDNF) levels in schizophrenia are indistinguishable from controls.. Neurosci Lett.

[52] Lee BH, Kim YK (2009). Increased plasma brain-derived neurotropic factor, not nerve growth factor-Beta, in schizophrenia patients with better response to risperidone treatment.. Neuropsychobiology.

[53] Sen S, Duman R, Sanacora G (2008). Serum brain-derived neurotrophic factor, depression, and antidepressant medications: meta-analyses and implications.. Biol Psychiatry.

[54] Brietzke E, Kauer-Sant'anna M, Teixeira AL, Kapczinski F (2009). Abnormalities in serum chemokine levels in euthymic patients with bipolar disorder.. Brain Behav Immun.

[55] Teixeira AL, Reis HJ, Nicolato R, Brito-Melo G, Correa H (2008). Increased serum levels of CCL11/eotaxin in schizophrenia.. Prog Neuropsychopharmacol Biol Psychiatry.

