# Supplementary material for: Plasma Protein Biomarkers for Depression and Schizophrenia by Multi Analyte Profiling of Case-Control Collections
Source: PLoS One. 2010 Feb 11;5(2):e9166. doi: 10.1371/journal.pone.0009166 (PMC2820097; doi:10.1371/journal.pone.0009166)
Supplement: Table S1 — List of markers assessed by the multi-analyte panel MAP 1.5 and their Least Detectable Dose. (0.11 MB PDF) [file pone.0009166.s001.pdf]

TABLE S1: list of markers assessed by the multi-analyte panel MAP 1.5

MAP1.5 analytes	units	Least Detectable Dose	Reference EDTA plasma concentration	MAP1.5 analytes	units	Least Detectable Dose	Reference EDTA plasma concentration
1. α-1 Antitrypsin	mg/mL	0.011	1.76	41. IL-7	pg/mL	53.4	70.34
2. Adiponectin	ug/mL	0.2	3.63	42. IL-8	pg/mL	3.51	7.02
3. α-2 Macroglobulin	mg/mL	0.061	0.22	43. IL-10	pg/mL	15.4	17.36
4. AFP	ng/mL	0.43	1.91	44. IL-12p40	ng/mL	1.22	1.3
5. Apolipoprotein A1	mg/mL	0.0066	0.35	45. IL-12p70	pg/mL	93.6	73.44
6. Apolipoprotein CIII	ug/mL	2.7	89.6	46. IL-13	pg/mL	56.9	92.78
7. Apolipoprotein H	ug/mL	8.8	255.2	47. IL-15	ng/mL	1.28	1.96
8. β-2 Microglobulin	ug/mL	0.013	2	48. IL-16	pg/mL	66.2	611.3
9. BDNF	ng/mL	0.029	2.46	49. IL-18	pg/mL	54	nd
10. Complement 3	mg/mL	0.00526	1.35	50. Insulin	uIU/mL	0.859	6.11
11. CA 125	U/mL	4.22	6.86	51. Leptin	ng/mL	0.103	8.65
12. CA 19-9	U/mL	0.246	1.61	52. Lipoprotein (a)	ug/mL	3.7	290
13. Calcitonin	pg/mL	6.04	<12	53. Lymphotactin	ng/mL	0.378	0.3
14. CEA	ng/mL	0.841	0.93	54. MCP-1	pg/mL	52	84.5
15. CK-MB	ng/mL	0.42	<1.1	55. MDC	pg/mL	13.9	326.4
16. CRP	ug/mL	0.00153	8.27	56. MIP-1 α	pg/mL	13	6.54
17. EGF	pg/mL	7.35	3.6	57. MIP-1 β	pg/mL	37.9	81.7
18. ENA-78	ng/mL	0.0755	0.39	58. MMP-2	ng/mL	150	278
19. Endothelin-1	pg/mL	7.18	8.97	59. MMP-3	ng/mL	0.2	<1.8
20. Eotaxin	pg/mL	41	36.1	60. MMP-9	ng/mL	37.2	65.49
21. Erythropoietin	pg/mL	166	<284	61. Myoglobin	ng/mL	1.05	10.71
22. FABP	ng/mL	2.99	1.13	62. PAI-1	ng/mL	0.9	33.38
23. Factor VII	ng/mL	1.02	276.2	63. PAP	ng/mL	0.0342	0.21
24. Ferritin	ng/mL	1.4	181.8	64. PSA, Free	ng/mL	0.0233	0.24
25. FGF basic	pg/mL	98	84.9	65. RANTES	ng/mL	0.0483	14.95
26. Fibrinogen	mg/mL	0.00983	3.66	66. Serum Amyloid I	ug/mL	0.0575	34.13
27. HGH	ng/mL	0.133	3.26	67. Stem Cell Factor	pg/mL	55.6	60.1
28. GM-CSF	pg/mL	57.4	53.32	68. SGOT	ug/mL	3.72	14.58
29. GST	ng/mL	0.404	0.39	69. TBG	ug/mL	0.341	75.38
30. ICAM-1	ng/mL	3.15	117.9	70. Tissue Factor	ng/mL	0.841	<2.4
31. IgA	mg/mL	0.00835	3.35	71. TIMP-1	ng/mL	8.39	92.9
32. IgE	ng/mL	14	109.4	72. TNF RII	ng/mL	2	77.06
33. IgM	mg/mL	0.0151	1.47	73. TNF-α	pg/mL	3.99	4.58
34. IL-1 α	ng/mL	0.163	0.25	74. TNF-β	pg/mL	45.6	21.95
35. IL-1 β	pg/mL	1.46	3.86	75. Thrombopoietin	ng/mL	3.23	4.2
36. IL-2	pg/mL	60	26.17	76. TSH	uIU/mL	0.028	1.38
37. IL-3	ng/mL	0.173	1.03	77. VCAM-1	ng/mL	2.6	518.8
38. IL-4	pg/mL	104	44.67	78. VEGF	pg/mL	7.5	122
39. IL-5	pg/mL	32.5	37.16	79. vWF	ug/mL	0.4	18.97
40. IL-6	pg/mL	12.2	<25				
