# Supplementary figures and images for: Plasma Protein Biomarkers for Depression and Schizophrenia by Multi Analyte Profiling of Case-Control Collections
Source: PLoS One. 2010 Feb 11;5(2):e9166. doi: 10.1371/journal.pone.0009166 (PMC2820097; doi:10.1371/journal.pone.0009166)

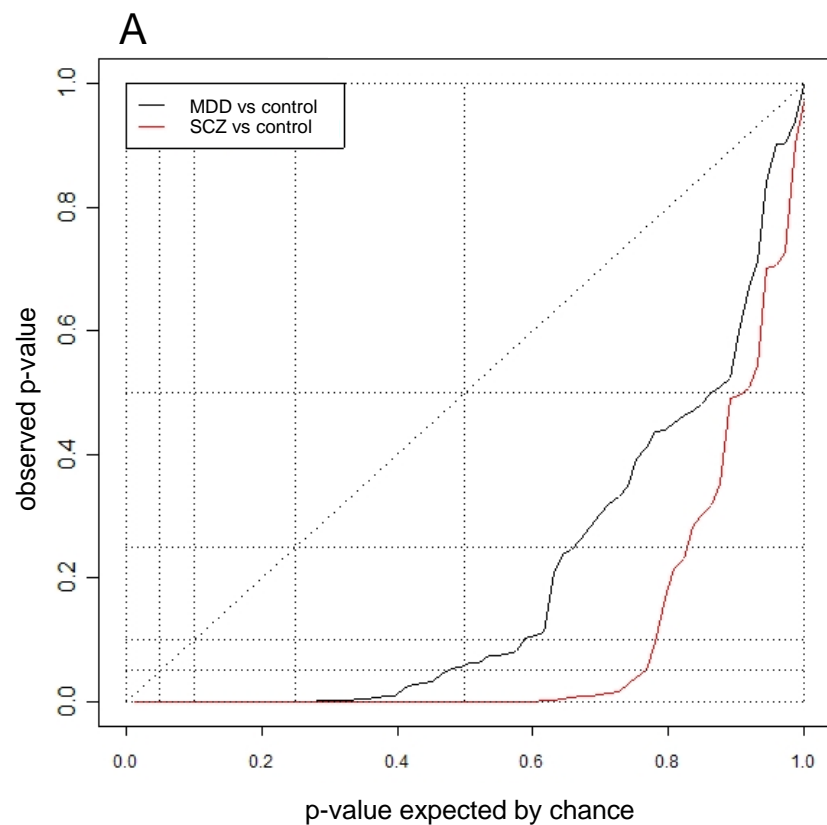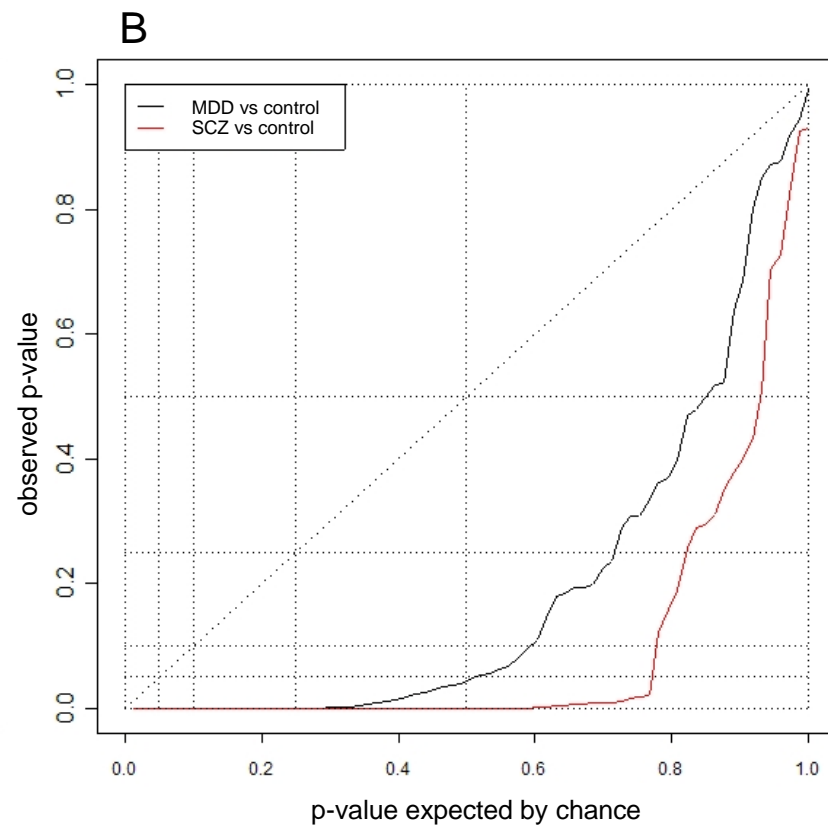

Supplement: Figure S1 — Non-parametric (a) and rank transformation (b) results referenced against analysis of variance with minimum value imputation. The observed p-values strongly deviate from the expected p-values that would be expected by chance, both for depression (MDD) and schizophrenia (SCZ) samples. (0.27 MB PDF) [file pone.0009166.s004.pdf]
