# Supplementary material for: Plasma Protein Biomarkers for Depression and Schizophrenia by Multi Analyte Profiling of Case-Control Collections
Source: PLoS One. 2010 Feb 11;5(2):e9166. doi: 10.1371/journal.pone.0009166 (PMC2820097; doi:10.1371/journal.pone.0009166)
Supplement: Figure S2 — Simple p values vs p values after covariate analysis. Correlations between simple p values and p values after inclusion of covariates in the analysis. (0.21 MB PDF) [file pone.0009166.s005.pdf]

FIGURE S2: simple p values vs p values after covariate analysis

Correlations between simple p values and p values after inclusion of covariates in the analysis

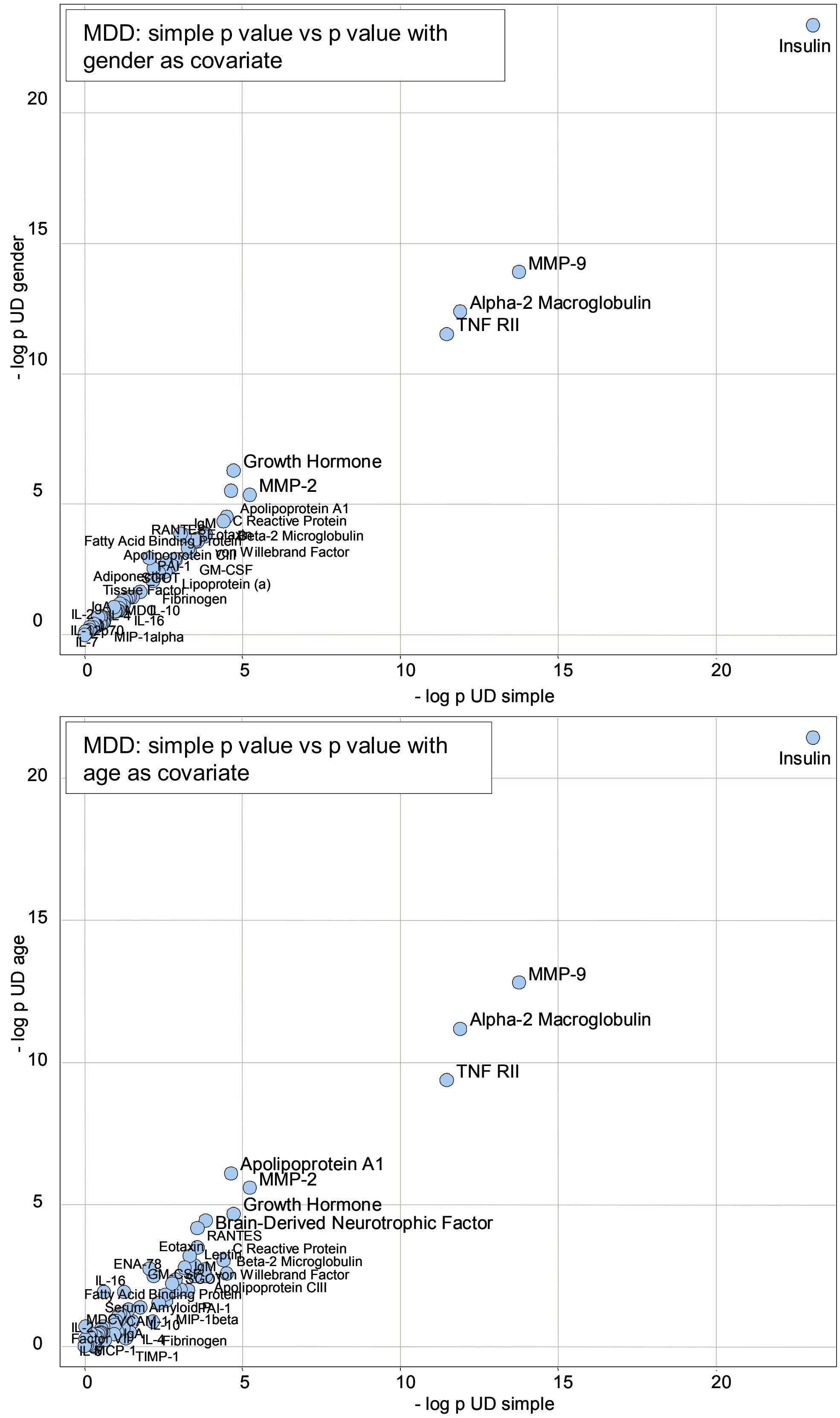

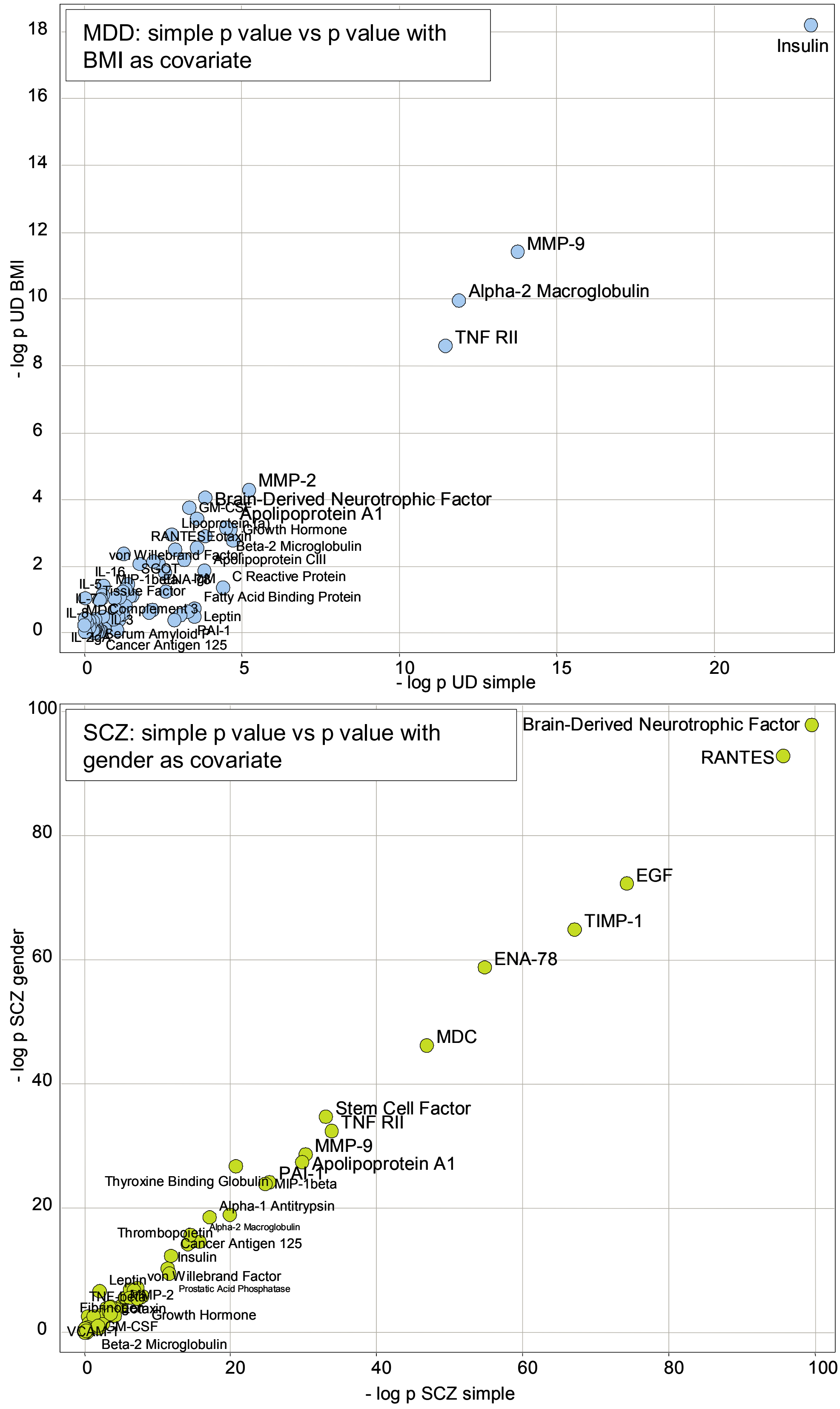
MDD: simple p value vs p value with BMI as covariate
- log p UD BMI
- log p UD simple
Insulin
MMP-9
Alpha-2 Macroglobulin
TNF RII
MMP-2
Brain-Derived Neurotrophic Factor
Apolipoprotein A1
Growth Hormone
Beta-2 Microglobulin
Apolipoprotein CIII
C Reactive Protein
Fatty Acid Binding Protein
Leptin
PAI-1
Cancer Antigen 125
Serum Amyloid P
Tissue Factor
von Willebrand Factor
SGOT
RANTES
Eotaxin
GM-CSF
IL-16
IL-5
IL-13
SCZ: simple p value vs p value with gender as covariate
- log p SCZ gender
- log p SCZ simple
Brain-Derived Neurotrophic Factor
RANTES
EGF
TIMP-1
ENA-78
MDC
Stem Cell Factor
TNF RII
MMP-9
Apolipoprotein A1
PAI-1
MIP-1beta
Thyroxine Binding Globulin
Alpha-1 Antitrypsin
Alpha-2 Macroglobulin
Thrombopoietin
Cancer Antigen 125
Insulin
von Willebrand Factor
Leptin
Prostatic Acid Phosphatase
MMP-2
Growth Hormone
GM-CSF
VCAM-1
Beta-2 Microglobulin

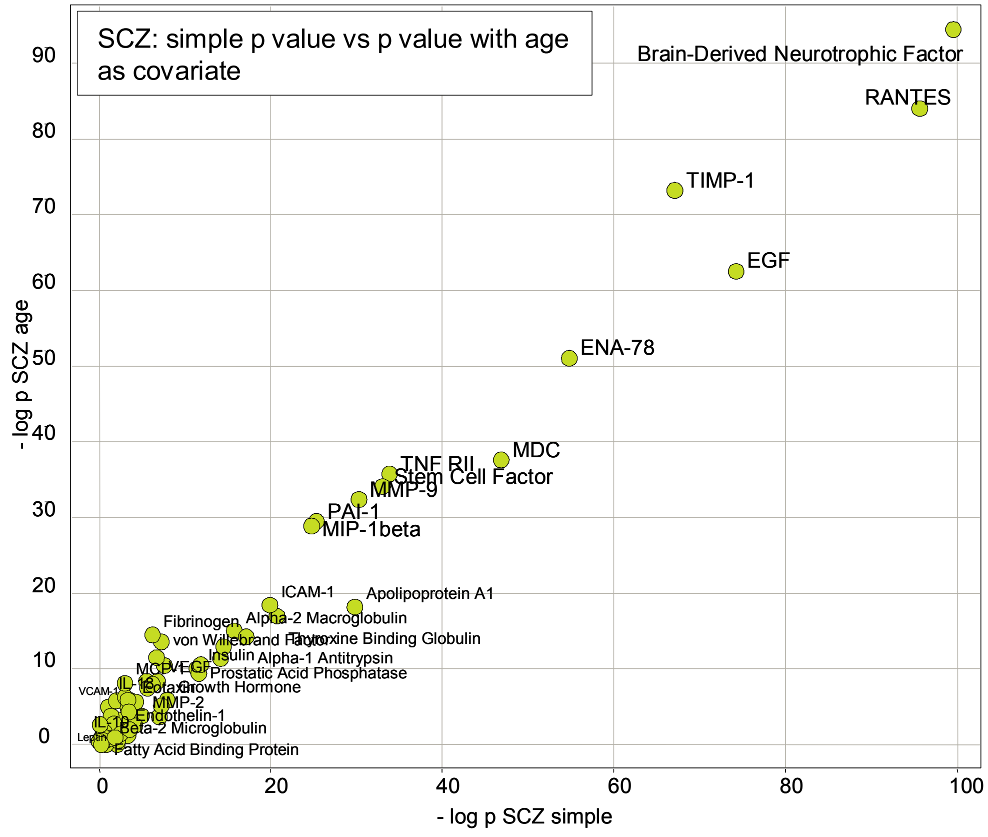

SCZ: simple p value vs p value with age as covariate
Brain-Derived Neurotrophic Factor
RANTES
TIMP-1
EGF
ENA-78
MDC
TNF RII
Stem Cell Factor
MMP-9
PAI-1
MIP-1beta
ICAM-1
Apolipoprotein A1
Fibrinogen
Alpha-2 Macroglobulin
von Willebrand Factor
Thyroxine Binding Globulin
Insulin
Alpha-1 Antitrypsin
VEGF
Prostatic Acid Phosphatase
Growth Hormone
MMP-2
Endothelin-1
Beta-2 Microglobulin
Fatty Acid Binding Protein
- log p SCZ age
- log p SCZ simple
