# Supplementary material for: Plasma Protein Biomarkers for Depression and Schizophrenia by Multi Analyte Profiling of Case-Control Collections
Source: PLoS One. 2010 Feb 11;5(2):e9166. doi: 10.1371/journal.pone.0009166 (PMC2820097; doi:10.1371/journal.pone.0009166)
Supplement: Figure S3 — Effect of dietary confounders on insulin levels (depression samples). (0.10 MB PDF) [file pone.0009166.s006.pdf]

FIGURE S3: Effect of dietary confounders on insulin levels (depression samples)

Further analyses have been carried out to assess potential dietary and site confounder that might have affected the finding of insulin elevation in depressed patients. As it can be seen in the graph below, a potential confounder is the elapsed time between sampling and last meal that was differing across study sites and subject groups, which we have used as a covariate in additional exploratory analyses. From the clinical chemistry, data on glucose levels were also available, and the insulin data were adjusted with glucose levels (to take into account the glucose-insulin homeostasis and compensate for potential post-prandial effects). The data obtained indicate that adjustment for elapsed time from meal and for glucose levels reduces the relative insulin increase from 122.4% to 80.0% or 73%, though the data remain highly significant (p<0.001).

Relative increases in insulin levels after adjustment with additional covariates

Insulin depressed vs controls	122.4 % difference (p<.0001)
Insulin adjusted for time since last meal	80.0 % difference (p<.0001)
Insulin adj. for TSM, gender, glucose level	73.0 % difference (p<.0001)

Elapsed time between sampling and last meal across study sites and patient groups, for depression samples

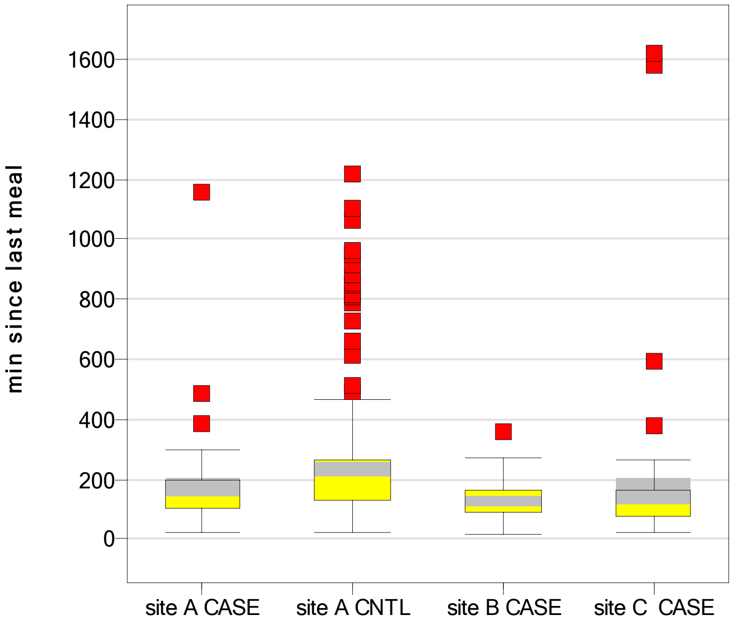

	site A CASE	site A CNTL	site B CASE	site C CASE
Mean	171.1	232.7	126.4	159.7
StdDev	138.2	188.3	59.0	226.8
Count	77	252	66	94

site c/c
