# Supplementary material for: Plasma Protein Biomarkers for Depression and Schizophrenia by Multi Analyte Profiling of Case-Control Collections
Source: PLoS One. 2010 Feb 11;5(2):e9166. doi: 10.1371/journal.pone.0009166 (PMC2820097; doi:10.1371/journal.pone.0009166)

FIGURE S4: Results from Random Forest (RF) algorithm
Discriminative models based on cross validation with a training set and a test set generated by RF and relative ROC curves.

To further support the findings obtained by PCA and PLS-DA, we have run a complementary analysis based on a random forests (RF, see Breiman 2001, Machine Learning 45: 5–32). By generating populations (a.k.a. forests) of decision trees, the RF approach provided a completely independent view of the data which was tolerant of data scale issues (c.f. data transformation) characteristic of the assay platform. For each node of every tree in each random forest, a total of three randomly selected variables were considered, although the model appeared generally stable to the choice of this parameter. Furthermore, to illustrate the predictive power of this dataset, this work was also cross validated with 15 random independent draws into training and test set (75% and 25%).
ROC curves (based on the ability to classify the test set) are presented for each individual random draw (grey lines) together with the average ROC derived from all draws.
Finally, the Mean Decrease Accuracy plots provide a measure of the importance or the contribution of each analyte to the discriminative model (i.e. to the last drawn random forest model for each comparisons). As it can be seen, the rank order of the analytes is similar to that obtained by PLS-DA.

See figures below

1. **Mean Decreased Accuracy from RF algorithm (MDD vs controls; SCZ vs controls).**

2. **ROC curves from RF calculations; from 15 random independent draws (MDD vs controls; SCZ vs controls).**

Mean Decreased Accuracy from RF algorithm (MDD vs controls).

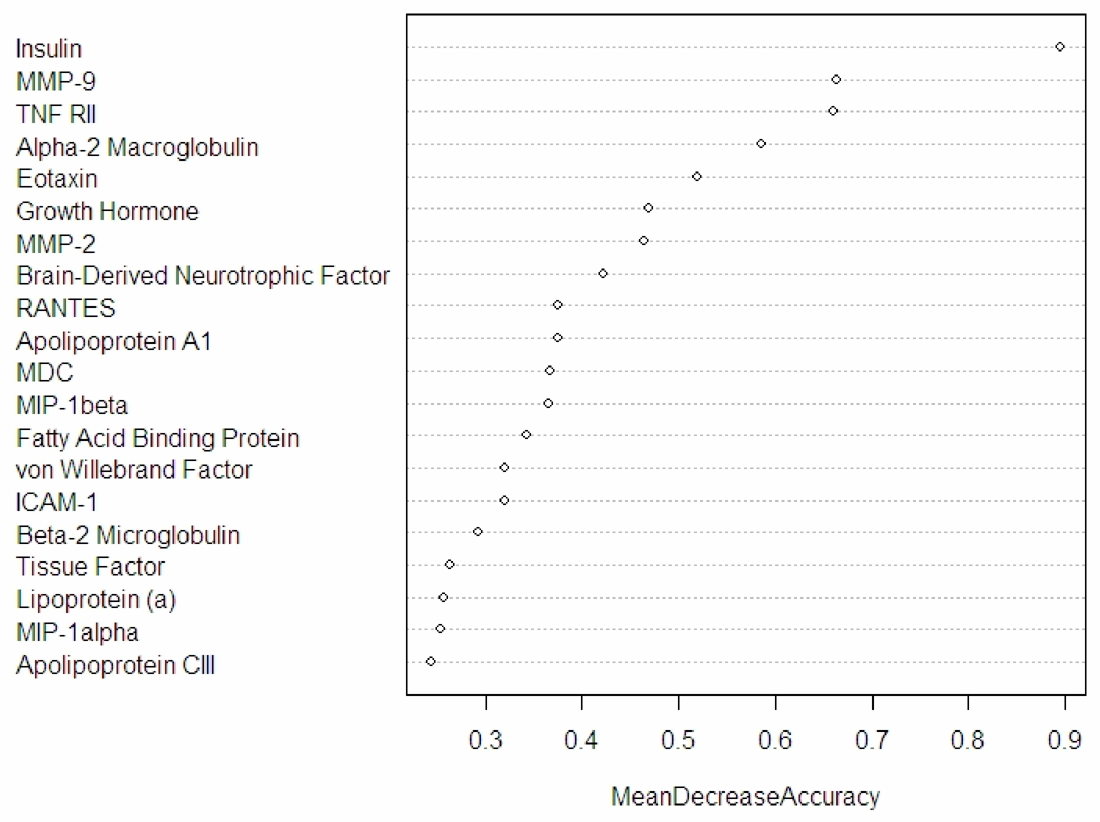

Mean Decreased Accuracy from RF algorithm (SCZ vs controls).

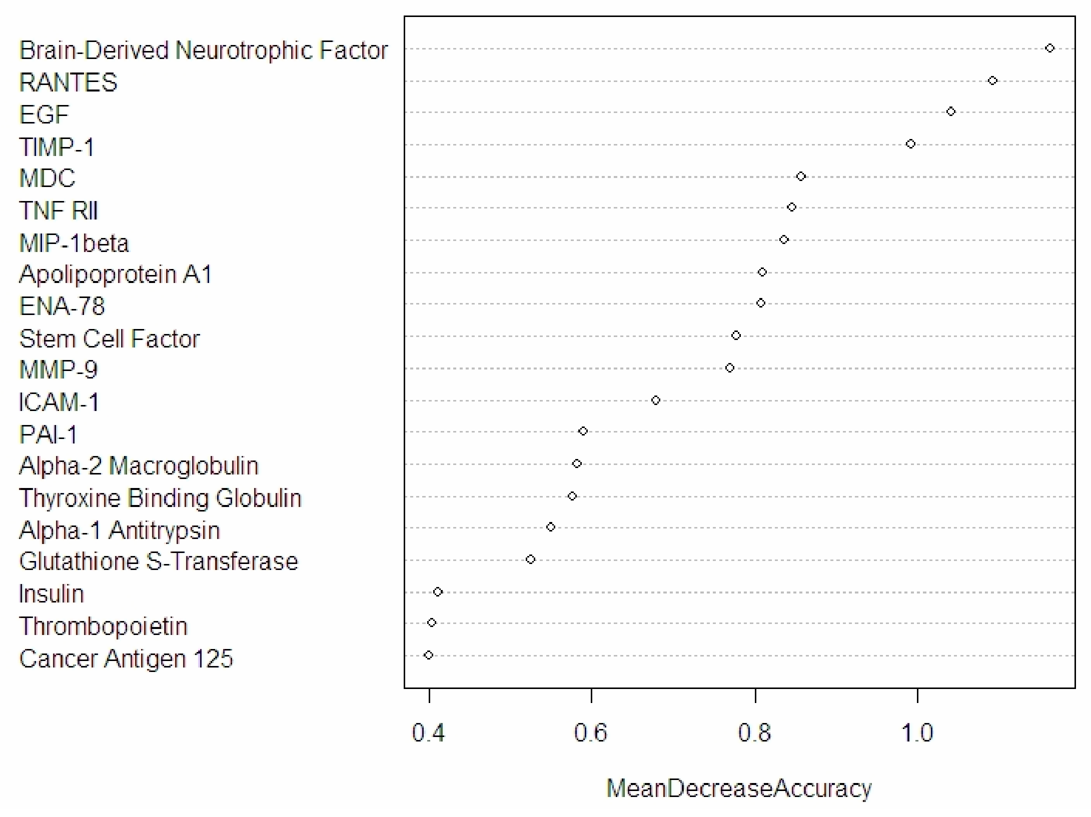

ROC curves from RF calculations; from 15 random independent draws (MDD vs controls).

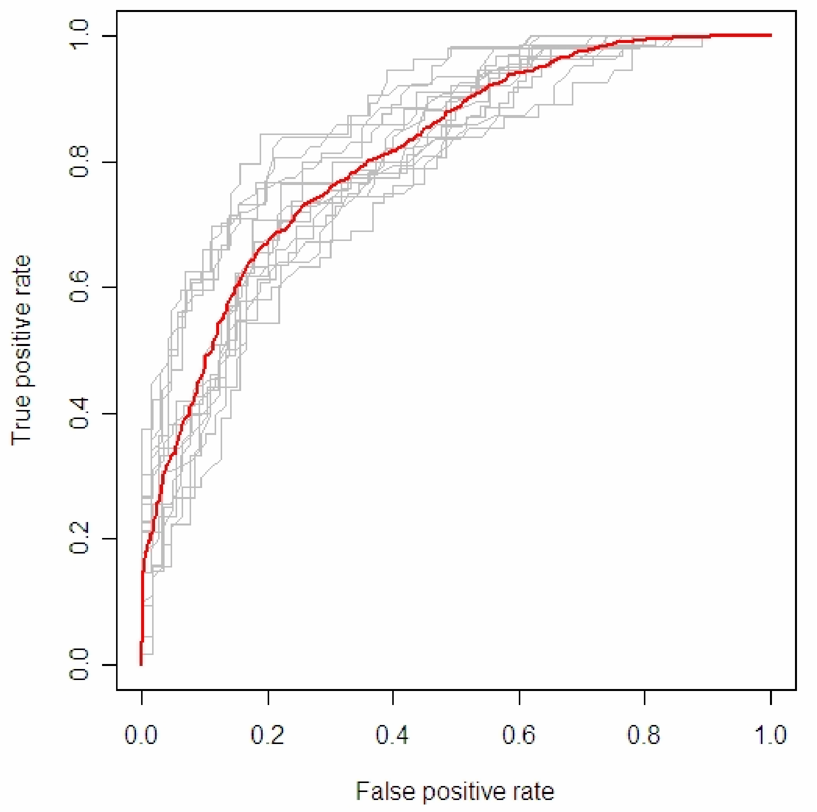

ROC curves from RF calculations; from 15 random independent draws (SCZ vs controls).

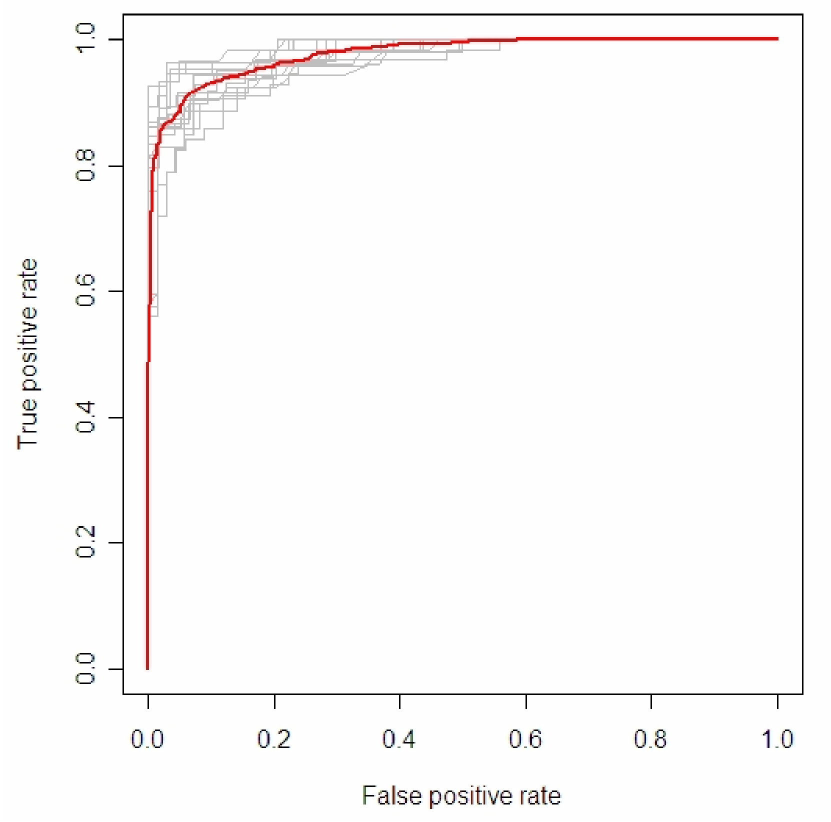

Supplement: Figure S4 — Results from Random Forest (RF) algorithm. Discriminative models based on cross validation with a training set and a test set generated by RF and relative ROC curves. (0.28 MB PDF) [file pone.0009166.s007.pdf]
