# Supplementary material for: Plasma Protein Biomarkers for Depression and Schizophrenia by Multi Analyte Profiling of Case-Control Collections
Source: PLoS One. 2010 Feb 11;5(2):e9166. doi: 10.1371/journal.pone.0009166 (PMC2820097; doi:10.1371/journal.pone.0009166)
Supplement: Figure S5 — Correlation with clinical severity (schizophrenia samples). Results from Spearman's correlation test run between protein levels in the plasma and PANSS value for schizophrenic patients. The central dots are the correlations, and the horizontal lines their 95% confidence intervals. The twenty analytes with the highest correlation are shown. (0.07 MB PDF) [file pone.0009166.s008.pdf]

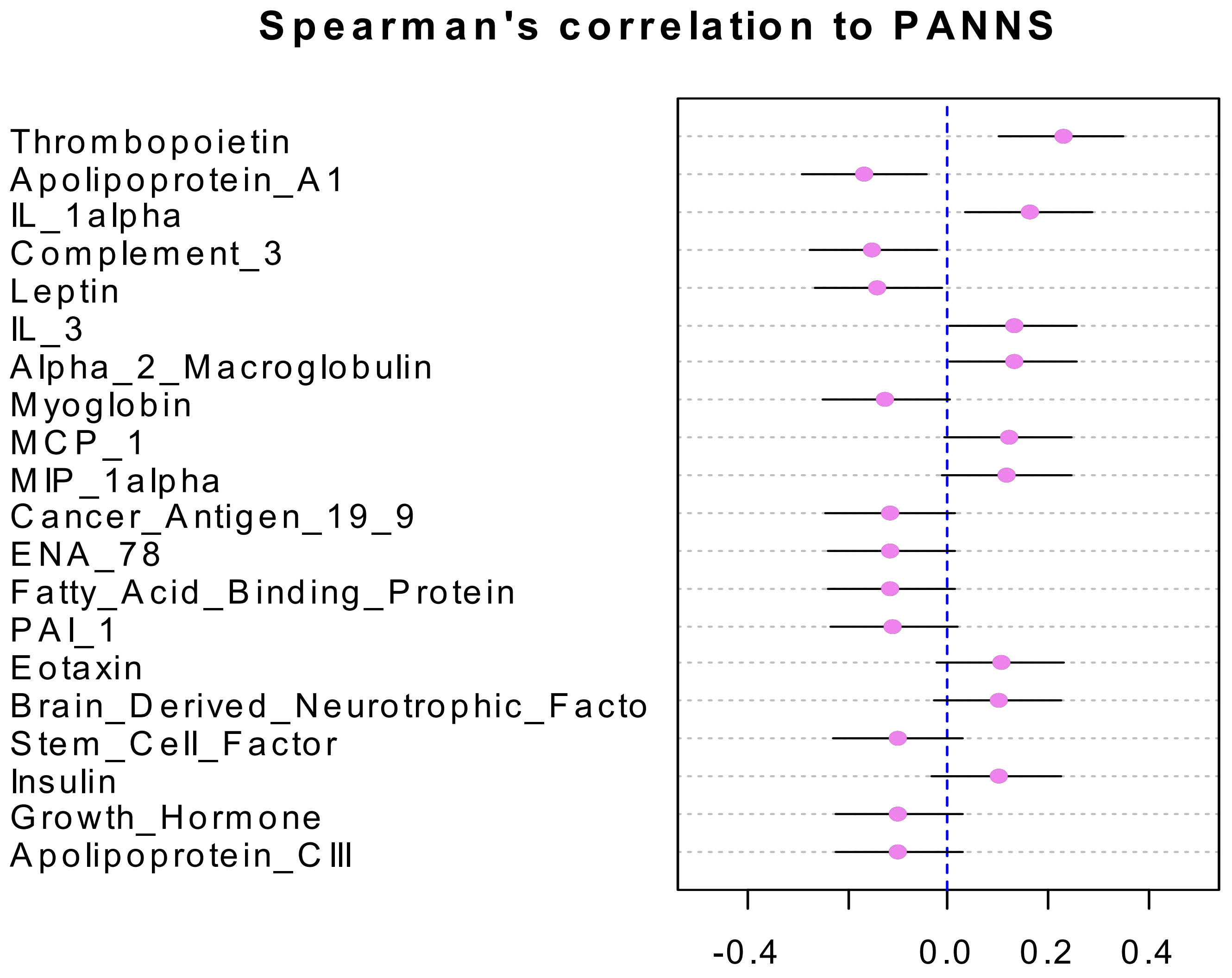
Spearman's correlation to PANNS
Thrombopoietin
Apolipoprotein_A1
IL_1alpha
Complement_3
Leptin
IL_3
Alpha_2_Macroglobulin
Myoglobin
MCP_1
MIP_1alpha
Cancer_Antigen_19_9
ENA_78
Fatty_Acid_Binding_Protein
PAI_1
Eotaxin
Brain_Derived_Neurotrophic_Facto
Stem_Cell_Factor
Insulin
Growth_Hormone
Apolipoprotein_CIII
-0.4
0.0
0.2
0.4
